# An exploration of teacher and school-based nurse perceptions of current HPV education offered to students 15–16 years old in post-primary schools in Northern Ireland, UK

**DOI:** 10.1371/journal.pone.0311651

**Published:** 2024-10-07

**Authors:** Terri Flood, Dr. Marian McLaughlin, Dr. Iseult Wilson, Ciara M. Hughes

**Affiliations:** 1 School of Health Sciences, Ulster University, Londonderry, Derry, United Kingdom; 2 School of Psychology, Ulster University, Londonderry, Derry, United Kingdom; 3 School of Nursing and Midwifery, Queen’s University Belfast, Belfast, United Kingdom; University of Udine: Universita degli Studi di Udine, ITALY

## Abstract

**Introduction:**

Human papillomavirus virus (HPV) is highest among young adults 15–24 years old. High-risk strains are responsible for the development of cancers including cervical, vaginal, vulvar, anal, oropharyngeal and penile. Despite HPV school-based vaccination programmes in the UK, HPV vaccination uptake rates continue to fluctuate due to misinformation and vaccine hesitancy post COVID-19. The aim of this study is to explore perceptions of post-primary school teachers and nurses regarding the current HPV education provision and the need to provide HPV education to students 15–17 years old when they are most likely to be becoming sexually active.

**Methods:**

A qualitative study was conducted using online semi-structured interviews between February-August 2022 with post-primary teachers and nurses in Northern Ireland, UK. Stratified random sampling was used to contact schools to recruit participants. Recruitment continued until data saturation was reached. Braun & Clarke’s six-phase framework for reflexive thematic analysis was used to analyse the data.

**Results:**

Twelve teachers and six nurses participated in the study. Four themes arose based on the analysis including 1) the importance of HPV education 2) self-consent to the HPV vaccine 3) design of the HPV education and 4) delivery of the HPV education. Identified barriers to implementation of HPV education included lack of parental education, religion and the conservative culture of Northern Ireland.

**Discussion:**

Participants perceived HPV education to be poor or non-existent in their schools but placed high importance on this education. They indicated that a non-judgemental health professional would be the ideal person to deliver interactive HPV education as part of a mandated spiral curriculum.

**Conclusion:**

HPV education at 15–17 years old provides students with an opportunity to learn about their HPV risk, their HPV vaccination status and an opportunity to self-consent to the HPV vaccine. The Education Authority and Department of Health should support health professionals to deliver consistent robust HPV health information to students of this age.

## Introduction

Human papillomavirus (HPV) is the most prevalent sexually transmitted infection (STIs) in the world [1] and is highest in young people aged 15–24 years old [2]. HPV is estimated to be the cause of over 90% of cervical cancers, 90% of anal cancer, 65% of vaginal cancers, 50% of vulvar cancers, 45-90% of oropharyngeal cancers and 50% of penile cancers [2–4]. It is a unique STI as, unlike other STIs, it can be prevented through vaccination [2]. The optimal age for administration of the HPV vaccination is 11–13 years old, prior to initiation of sexual activity [2].

In European countries, HPV vaccination has been gradually introduced in national immunisation programmes since 2007; however, many European countries including France and Germany, face sub-optimal levels of HPV vaccine uptake [5]. Conversely, the UK has achieved remarkably high HPV vaccination rates since the introduction of a school-based vaccination programme in 2008 [5, 6]. This school-based programme was initially offered to females 12–13 years old and since 2019 has been extended to include males of the same age [7]. Specialist public health nurses, called immunisation nurses, deliver this programme in schools [8] and provide a leaflet for parents to read prior to consenting their child for HPV vaccination [9]. Since the introduction of the HPV immunisation programme, cervical cancer rates in the UK have fallen by 87% [10] with similar results observed in Australia [11]. Decreased rates of HPV acquirement have also been reported in males who received the HPV vaccination in Australia [12]. By June 2020, 107 of the 194 World Health Organisation (WHO) Member States had introduced national HPV vaccination programmes, with nearly 60% of programmes being delivered via a school-based programme [13].

Pre-COVID-19, 82–85% of females received at least one HPV vaccine in the 1^st^ year of post-primary school in the 2018–2019 academic school year in the UK [14–17]. Despite the majority of legal COVID-19 restrictions ending in March 2022 [18], based on the latest available national statistics from 2021–2022, uptake rates decreased significantly to between 70–78% of females receiving at least one HPV vaccine in the first year of post-primary school throughout the UK [15, 19–21]. Male HPV vaccination were approximately 7% lower than females for this same time period [15, 19–21]. Similar reduced HPV vaccination uptake in young males (compared to young females) has also been observed in other countries including Switzerland [22] and the US [23]. This sharp decline in HPV vaccination is consistent with that observed in immunisation rates globally since the COVID-19 pandemic [24, 25]. Since 2019, HPV vaccination coverage globally dropped by fifteen percent, which is one of the worst declines of any vaccine during the pandemic [26].

Additionally, many European countries including France, Republic of Ireland and Denmark have seen large fluctuations in HPV vaccination uptake rates over the past decade as anti-vaccination movements have contributed to parental misinformation regarding HPV vaccine safety [27]. Japan also experienced a sharp decline in HPV vaccination in the 2000s due to extreme negative mass media resulting in the government suspending proactive recommendations of HPV vaccines; in 2020, Japan still had one of the lowest HPV vaccine coverages among high-income countries [28]. Overall, despite the availability of safe and effective human papillomavirus (HPV) vaccines, vaccination uptake remains relatively low in many high-income countries including the U.S, Italy, the Netherlands and Germany and very low in most middle-income and low-income countries [29].

Despite parental concerns, numerous randomised controlled trials (RCTs) have consistently confirmed the vaccine to be highly safe with no serious adverse events reported [2, 5]. Given that parents are the primary decision-makers regarding their child’s HPV vaccination uptake, it is unsurprising that students demonstrate poor knowledge of HPV and their risk of acquiring HPV, regardless of their vaccination status [30]. This knowledge deficit has been demonstrated to be particularly high in males, though young people’s knowledge regarding the link between HPV and cancers other than cervical cancer is generally poor for all genders [31–33].

Parental knowledge of HPV has been found to range from low to moderate [34–37] with higher deficits being reported in association with HPV in males [33]. Importantly, parental knowledge is strongly associated with intention to vaccine [38].

At 15–16 years old, information regarding HPV is likely to be much more relevant to adolescents, who may be considering or are already engaged in sexual relationships. The average age of first sexual intercourse in most developed regions of the world including Europe, Australia and the US is 15–18 years old [39–43] and therefore educating students of this age regarding HPV transmission and their HPV vaccination risk is important. Additionally, many adolescents engage in oral sex prior to penetrative sex [44] with use of condoms during oral sex being low [45].

Consequently, providing additional HPV education and assess to HPV vaccination at 15–16 years old, could provide an additional opportunity for students to self-consent to the HPV vaccine at this time without the need for parental consent [46]. Some adolescents may wish to be vaccinated contrary to their parents’ views.

The age of consent for vaccination programmes throughout the world varies considerably though in countries like the UK, Canada and Sweden and some parts of the US and Australia, adolescents are able to override their parents decisions if they are deemed able ‘to understand the consequences of the decision to vaccinate’ (page 418) [47]; this is termed Gillick competence [47, 48]. A person under 16 years old can be considered Gillick competent if they demonstrate a clear understanding of the HPV vaccine and potential consequences of HPV vaccination [48]. However, despite adolescent consent for vaccines being supported by the ethical principle of autonomy and developmental research on adolescents, in practice, self-consent for vaccines is very uncommon [47]. Fisher et al.’s mixed-methods evidence synthesis found that support for self-consent for HPV vaccination varied considerably among teachers and nurses, with most of them refusing to facilitate self-consent due to fear related to repercussions from parents [47]; a finding consistent with other similar research [49, 50]. Chantler et al.’s survey data found that only 32% of parents discussed vaccination with their teenager and parental awareness about the option for adolescents to self-consent for vaccination was limited [51]. Additionally Woods et al. noted that approaches to self-consent in the absence of parental consent vary considerably throughout schools in the UK [49].

To date there has been limited research exploring the views of teachers and nurses involved in the delivery of the HPV immunisation programme, especially related to their views of student self-consent [49].

Given this gap in research, the recent global declines and fluctuations in HPV vaccination and the lack of clarity among stakeholders regarding the rights of middle adolescents to self-consent to the HPV vaccination, further investigation into this area of sexual health is warranted.

Consequently, this study aimed to capture the perspectives of post-primary school teachers and school-based nurses regarding current HPV education, and whether they feel that more education is needed for students in year 12 in Northern Ireland (NI), UK. In NI, year 12 is the fifth year of post-primary education when students are typically 15–16 years old [52]. Teachers and nurses attitudes to self-consent to the HPV vaccine were also captured as part of this study.

## Methods

### Study design

A qualitative descriptive design was deemed the most appropriate to address the research aims as this methodology recognises the subjective nature of the problem and the different experiences that participants may have based on their experience and personality [53]. This qualitative approach is particularly useful when evaluating approaches to healthcare where ‘the focus is not on increasing theoretical or conceptual understanding, but rather contributing to change and quality improvement’ (p444) [53].

Interviews were chosen as the qualitative research method as they are an ideal format to gather information about the views and experiences of teachers and nurses. This type of study design encourages open, detailed dialogue and rapport building between the interviewer and interviewee [54]. In this role the interviewer supports open discussion by acting as a nonjudgmental, respectful, and active listener during the process [54]. Semi-structured interviews were utilised in this study; they are the most common types of interviews used in research and health settings and promote in-depth conversations [55]. See S1 File in Supporting Information section.

### Participants

Participants were recruited into the study from 1^st^ February-2022 to 1^st^ August 2022. Using an online randomiser [56], stratified random sampling was used to contact schools from the Department of Education’s list of 193 post-primary schools in NI [57]. Stratification was chosen to ensure inclusion of schools from a variety of religious and socioeconomic backgrounds with single and mixed genders, as these variables have all been found to influence HPV vaccine uptake [58–61]. E-mails were addressed to the Head of School asking them to distribute details of the study to appropriate teachers and nurses within their school. Qualified teachers were eligible to participate in the study if they were; a science teacher; a teacher of relationship and sexuality education (RSE); Vice Principal or a Principal. Qualified teachers were excluded if they were not currently actively teaching in post-primary schools in NI. Teachers and teaching assistants who were not registered with The General Teaching Council for Northern Ireland were also excluded from the study.

School nurses were eligible to participate in the study if they were employed within a post-primary school and had qualified as a nurse. Auxiliary nurses without a nursing qualification were not eligible to participate.

### Consent and ethics

Teachers and nurses were asked to contact the research team if they were interested in participating in the study and were provided with further information via a Participant Information Sheet after their initial contact. They were encouraged to contact the research team with questions prior to providing consent.

Consent was completed via Qualtrics [62], a secure online survey platform; participants also provided basic demographic information at this time. After completion of consent, online video interviews via Microsoft teams, were arranged with each of the participants.

Ethical approval for this project was granted by the NHS Research Ethics Committee in September 2020 (ID:287358).

### Methodological rigour

The COREQ (COnsolidated criteria for REporting Qualitative research) Checklist was used to ensure methodological rigour; there are four Domains which need to be addressed within this checklist [54].

Domain 1 is concerned with the credentials of the research team and reflexivity [54]. All four researchers were lecturers within the schools of health sciences, nursing and psychology within a university and identified as female. Three members of the research team held post-graduate doctorate degrees and had vast experience with both qualitative and quantitative research design and delivery. The fourth researcher, who was working toward completing their postgraduate doctorate degree, held a post-graduate Qualitative Practical Skills Workshop Certificate and facilitated all online interviews. This researcher was also experienced in conducting interviews and qualitative research. To verify the integrity of the interview process, a second researcher reviewed the first two interviews to ensure that the interview process was transparent, reflexive and unbiased. Reflexivity highlights the importance of the researcher’s background and prior perceptions in potentially influencing and biasing the research process [63]. Peer evaluation provided by the second researcher enabled the team to collaboratively reflect upon the influence of their own characteristics and experiences to ensure that all interviews were facilitated in an unbiased, neutral manner. Peer evaluation is considered an effective tool in ensuring rigour and reducing bias in qualitative research [64]. None of the interviewees were known to the interviewer prior to the interviews, which also reduces potential bias.

Domain 2 of the COREQ checklist is concerned with the study design [54]. Participant selection is transparent and has been previously described. To increase the validity of interviews, an interview guide (see S1 File) was developed based on a systematic review of the literature [27] and feedback from prior and concurrent focus groups and interviews conducted by the research team within this project [65, 66]. Interviews with teachers were discontinued once data saturation was reached. Data saturation is considered the gold standard in qualitative research and occurs when no additional data is being added i.e. participants are repeating information already established in previous interviews [67]. For the teacher interviews, data saturation was reached after twelve interviews. This aligns to other qualitative studies which suggest that data saturation occurs after 9–17 interviews [68]. Unfortunately, due to the small number of school-based nurses in post-primary schools in NI, it was unclear whether data saturation was reached in this group. Time limitations on the project and access to post-primary schools post-COVID 19, resulted in the project being stopped before data saturation in this group could be confirmed. Transcripts were sent to participants for member checking to increase study credibility and confirmability [63].

Domain 3 is concerned with Data Analysis [54] and findings and is detailed within the following section.

### Data analysis

The video-recorded interviews were professionally transcribed and anonymised by the transcriber through the addition of a numerical value for each interview to replace the interviewee’s name. As recommended by the FORS Guides, anonymisation should be conducted at the time of transcription [69].

Braun & Clarke’s six-phase framework for reflexive thematic analysis was used to analyse the data [70, 71]. To ensure rigour, two of the researchers independently completed steps one to three. Step one involved ensuring strong familiarity with the interview content through watching the interview and re-reading the transcripts. Step two involved the researchers utilising NVivo® (QSR International Pty Ltd) to develop a codebook through line-by-line coding. Step three involved combining codes into appropriate categories and further into themes. During step four and five, the two researchers discussed and modified the categories and themes. While most discrepancies in the categories and themes were resolved between the two researchers, any unresolved disagreements were discussed with the other two members of the team until consensus was reached. The final themes are described in the results section (step 6).

Reflexivity is an important consideration in Braun & Clark’s more recent (2019) framework [71]. To reduce this potential influence, the two researchers involved in the analysis were from different academic backgrounds; one researcher from psychology and a second from radiotherapy. As a team, the researchers discussed and acknowledged how their preconceptions and assumptions could affect the analysis process.

While thematic analysis can be either scientific or interpretivist in nature, the analytical approach in this study was scientific, relying on reliable coding procedures to inductively create categories and themes [70]; this approach also reduces the potential for influence of personal opinions and perceptions.

## Results

Twelve teachers and six nurses participated in the study during the seven-month recruitment period. Interviews ranged from 25–60 minutes (average 44 minutes). See full demographic information in Table 1. While a choice of eight religious denominations were offered as options, participants all identified themselves as being either Catholic (n = 8; 44.4%), Protestant (n = 7, 38.9%), Christian (not Catholic or Protestant) (n = 2; 11.1%) or having no religion (n = 1, 5.6%). Fourteen options were provided for ethnicity but only two options were chosen; White Northern Irish (n = 16, 88.9%) and White Non-Northern Irish (n = 2, 11.1%). Fourteen of the eighteen participants (77.8%) identified as female.

**Table 1:** Demographic information of teachers and school-based nurses.

	Teachers		Nurses	
	N	%	N	%
**Age**				
20–34	2	16.7		
35–49	7	58.3		
50–69	3	25	6	100
**Gender**				
Male	4	33.3		
Female	8	66.7	6	100
Non-binary				
**Ethnicity**				
White Northern Irish	11	91.7	5	83.3
White Non-Northern Irish	1	8.3	1	16.7
**Religious Denomination**				
Protestant	4	33.3	3	50
Catholic	5	41.7	3	50
Christian (not Catholic or Protestant)	2	16.7		
None	1	8.3		
	Teachers only			
	N	%		
**Roles in school**				
Vice Principal	3	25		
Teach aspects of relationship and/or sexual health	4	33.3		
Biology teacher	7	58.7		
			Nurse only	
**What is your current nursing band e.g. band 5, band 6?**				
Band 5			3	50
Auxiliary			1	16.7
None			2	33.3
**How long have you been employed as a school-based nurse?**				
1–5 years			2	33.3
>5 years			4	66.7

Four themes emerged from the interviews

Importance of further HPV education

Self-consent to the HPV vaccine

Design of the HPV education

Delivery of the HPV Education

Each theme was further divided into subthemes. Table 2 highlights the key themes, subthemes and key language associated with each theme and subtheme.

**Table 2:** Summary of themes, subthemes and key language.

Theme	Subthemes	Key Language
1. Importance of further HPV education	1.1 Age-related sexual activity 1.2 Insufficient and inconsistent current HPV education 1.3 Barriers to HPV knowledge 1.4 Improvement in future sexual health outcomes and behaviours	• Motivation • School ethos • Role of School Nurse • Parents • Stigma • Social Media • Sexual practices • HPV screening
2. Self-consent to the HPV vaccine	2.1 Student maturity 2.2 Consent legislation and guidelines 2.3 Importance of parent-school relationship	• Boys • Gillick Consent • Education Authority (EA) • Department of Health (DoH) • Communication with parents
3. Design of the HPV education	3.1 Mandating HPV education through a spiral curriculum 3.2 Content of HPV educational intervention 3.3 Format of HPV educational intervention 3.4 Receipt of HPV vaccine	• Age • Timing • Non-vaginal sex • Sexual orientation • Consistent • Interactive • Terminology • Questions • Barriers
4. Delivery of the HPV Education	4.1 Characteristics of an ideal facilitator of HPV education 4.2 Teachers as facilitators of HPV educational intervention 4.3 Nurses as facilitators of HPV educational intervention	• Non-judgemental • Comfortable • Disclosure policy • Awkward • Embarrassment • Backlash • Experience • Knowledge • Training

### 1. Importance of further HPV education

Four subthemes emerged within this theme: 1) Age-related sexual activity; 2) Insufficient and inconsistent current HPV education; 3) Barriers to HPV knowledge; 4) Improvement in future sexual health outcomes and behaviours

#### 1.1 Age-related sexual activity

Several participants indicated that the percentage of students who are sexually active in any school will vary considerably and so the perceived importance of this education may vary depending on the ethos, religion, social status and gender of the school. Despite this perceived disparity, they spoke of the importance of empowering all students to enable them to make informed decisions regarding their own health through this education—particularly as some of the students in this age range are already sexually active and therefore this information was becoming very relevant.

*‘Certainly the sense that I get when we teach about sexual relationships is that a lot of these students are sexually active, definitely, already…I do think if the information was presented, a lot of them would take it up.’ (Teacher 1)*

#### 1.2 Insufficient and inconsistent current HPV education

Most participants indicated that the current HPV education in schools was inconsistent and insufficient. Many participants indicated that even if a student studies science GCSE, there is minimal to no education about HPV in NI post-primary schools, and the little there may be, would be dependent on the teacher.

*‘…the one minute we spend discussing it (HPV] in double award science, or triple award science at GCSE, that this is the human papilloma virus, this is where you get your vaccine for it, this is what it causes… then I’m away…there is no bigger conversation in school about this. And I don’t think our school is that much different from any other school.’ (Teacher 4)*

Some participants indicated that they felt that it is the duty of the school and themselves to ensure that they educate the students so that students are informed about safe sexual practices.

*‘…we are the ones who fail them. They didn’t know any better because we never taught them any better. And because it’s uncomfortable as an adult isn’t an excuse…you are supposed to be preparing them for life.’ (Teacher 4)*

Some participants noted that their school brought in external providers to address relationship and sexual development topics like pregnancy as part of their Professional Development (PD) programme. ‘Love for Life’, who is a Christian charity in NI [72], was most frequently mentioned and while they thought that these providers may briefly mention STIs, they did not think that HPV and other STIs were discussed in any detail in these sessions. They commented that sexuality education was inconsistent in schools depending on the ethos and/or affiliated religion of that school. They described STIs and safe sex as a stigmatised topic in schools preventing HPV and other STIs from being discussed.

*‘With most schools there’s an onus to make students aware of the dangers of promiscuity and not using protection…in Northern Ireland, being quite conservative, even that’s barely talked about in a lot of schools. So if they don’t talk about safe sex practices, then how are we going to talk about STDs or STIs that can result?’ (Teacher 1)*

It was clear that school nurses roles in sexual health education varied considerably depending on their own interest in this area and the ethos of the school. Nurses commented that, as a professional group, they were once more involved in sexuality education but that the school structure had changed, putting more responsibility on the teachers to arrange or teach this education.

*‘…there has been a lot lost over the years. Because the school nurses, the community school nurses used to come in and do puberty talks. That fell away years ago. And that was then left that the school had to sort that out.’ (Nurse 5)*

#### 1.3 Barriers to HPV knowledge

An overwhelming majority of participants felt students had poor knowledge of what HPV is and the purpose of HPV vaccination.

*‘You ask any of our students, what does HPV stand for? They couldn’t even tell you. Have you been vaccinated for it? And I guarantee they’ll say, I don’t know.’ (Teacher 7)*

Apart from the school curriculum, participants identified three other dominant factors which they felt influenced their students’ lack of knowledge of HPV and their HPV vaccination status; parents, society and internal motivation.

The majority of participants indicated that parental lack of education was a barrier to student knowledge as they are the primary decision-makers at the time of HPV vaccination. Some participants remarked on the importance of the school working with parents regarding HPV vaccination decision-making to reduce parental and student misinformation and help parents to have HPV-related conversations with their teenagers. Teachers and nurses spoke about how parents are often not happy with sexual health information being taught in school due to their personal values and/or religious beliefs and that parents were often unaware or in denial of the fact that their teenagers may be engaging in sexual activities. They highlighted the importance of framing HPV education as a health message rather than approaching it from an ethical or moral stance.

*‘ If you are coming at it from an ethical, moral framework, that can be very tricky. If you are coming at it from a health perspective, there’s not so much argument with that.’ (Teacher 3)*

Many participants indicated that parents would not want their children learning about transmission routes of HPV when they are 14–15 years old (year 11) but would be supportive of this education when they are 15–16 years old (year 12). They felt that most parents would be grateful that the school provides this education as parents find it difficult to have conversations about HPV transmission and sexual contact with their children, with many parents not having those conversations at all.

*‘These are conversations that we need to be having. And if the school is doing it for them, most parents are quite amenable to that, because it saves them having the conversation at home.’ (Teacher 2)*

Despite some of the parental barriers, participants remarked on the positive changes to both the Personal Development (PD) programme within schools and parents’ attitudes towards sexual health education, over the past decade.

*‘…when it came out first, it was a real…I don’t want my child to have it. But now people are getting more and more educated. And that’s what we need to do. Educate more and more people.’ (Nurse 1)*

All participants talked about the influence of society on students’ knowledge regarding HPV and their HPV vaccination status. They spoke about the conservative culture of NI and felt that sex was a stigmatised subject in this country—more so than in other countries. They felt that people often use religion to justify their conservative views, often associating STIs with promiscuity. One parent indicated to a teacher that ‘*only slappers get cervical cancer’*; slapper being an offensive slang word for a woman suggesting that she has a lot of sexual partners [73]. Participants spoke about how the NI culture influenced the school ethics and curriculum, influencing students’ knowledge of HPV.

*‘There’s this awful snobbery around sex education and especially here in Northern Ireland where we are supposed to be religious. And they hide behind these labels.’ (Nurse 3)*

The majority of participants highlighted the lack of information regarding HPV in media coverage including transmission routes, male HPV-associated cancers and long-term consequences of acquiring HPV. Over half of the participants referred to the influence of social media in promoting anti-vaccination propaganda and its contribution to the reduction in HPV vaccination rates in the Republic of Ireland. They also felt that this propaganda has increased since the COVID-19 pandemic and has had, and will continue to have, a negative impact on HPV vaccination rates.

*‘…some young people in recent times have heard vaccines are bad. And sometimes the argument has been very one sided, perhaps because the antivax group is so very vocal. So I think it education is going to become even more of a priority.’ (Teacher 3)*

*‘This past few years I have had the most anti-vaccination parents I’ve ever had in eighteen years*…*Covid has put paid to a lot of people even doing HPV*. *(Nurse 5)*

They felt that the open access that students have to social media platforms help them independently learn about sexual health and therefore they are more informed than previous adolescents at an earlier age. While these participants were aware of misinformation through social media platforms, they felt that students learned a lot of correct and relevant information in this way.

A few participants commented that before the age of 15–16, students are not motivated to know about HPV and find the association of HPV and genital warts disgusting.

*‘They [adolescents] don’t want to talk about the rest of it because, oh dear, warts, that sounds dirty and nobody wants to talk about that… .there’s a shame aspect… it causes a stigma for people when they start getting their smear tests, feeling dirty if they are HPV positive’ (Teacher 1)*

Other student motivating factors mentioned included students’ religious background, fear of needles and wanting to fit in with their peers.

#### 1.4 Improvement in future sexual health outcomes and behaviours

Several participants indicated that they felt that receiving this information at this stage would likely increase HPV screening uptake. They commented that without further education, female students may be under the impression that they do not need a cervical screening test due to having received the HPV vaccine. The majority of participants also expressed optimism that this HPV re-education would lead to safer sexual practices.

*‘…I would expect that if we educated them better, that their behaviours would change, whether that be that they reduce the amount of partners, which is what we hope for but also that they would increase how safe they are with a given partner.’ (Teacher 4)*

### 2. Self-consent to the HPV vaccine

Within this theme, three subthemes were identified; 1) Student maturity; 2) Consent legislation and guidance; 3) Importance of parent-school relationship

#### 2.1 Student maturity

All participants indicated that 16-year-old students would be mature enough to self-consent to the HPV vaccine and were aware of their legal rights to self-consent to vaccinations.

*‘I think they have to be given the information to be able to make that decision…it is their body… their choice…their future. It’s not really going to impact on their parents so much as it’s going to impact on them.’ (Teacher 3)*

The majority of participants supported 15-year-old students self-consenting though a few participants noted that some boys can be more immature and felt that an individual assessment might be the best option for this age group.

#### 2.2 Consent legislation and guidance

There was poor awareness among teachers and nurses of the legal rights of students to consent to vaccination under the age of sixteen (Gillick consent). Only two of the nurses demonstrated strong knowledge of Gillick consent and were experienced with implementing protocols around this process. Fourteen was generally deemed too young to self-consent. Even where a young person is deemed competent to consent, participants did not feel comfortable vaccinating any student without parental consent. They stressed that this could only change if clear procedures were established through the Education Authority (EA) and Department of Health (DoH) to support this initiative with clear consent guidelines for professionals involved and transparency between the school and parents if self-consent to HPV vaccination was offered alongside education for this age group.

*‘I would have thought you would have to wait to sixteen to do it [HPV education], so that they could consent themselves… then you don’t have that parental backlash at fifteen and saying, I still didn’t give permission. Because an awful lot of times, if they didn’t get it at twelve, it’s because mummy and daddy didn’t give parental permission…you could do it at the end of year twelve. At the end of year twelve they are all sixteen.’ (Teacher 4)*

‘…we allow them to go over to the family planning clinic. We don’t disclose to their parents about those sorts of things. I have taken children over myself who thought they were pregnant, to get pregnancy tests and that’s not disclosed to the parents. So absolutely they should be able to consent for a vaccine.’ (Teacher 9)

*‘Parents are a huge problem in schools*… *they are very demanding*…*I could imagine a parent being very annoyed if I tried to argue that a child had Gillick… they were competent*. *Yeah*, *I would find that difficult*.*’ (Nurse 5)*

#### 2.3 Importance of parent-school relationship

It was acknowledged that there would need to be a trusting relationship between the school and parents, and the students and their parents. As part of fostering a trusting relationship between students and their parents/guardians, several participants felt that providing an adequate time gap between when the students receive the HPV information and provided consent was very important, to enable their parents to look at the information and have a conversation with their teenager about their decision. They also recognised that these target parents were the same parents who did not want their child vaccinated previously so are the most challenging parents to convince of the benefits of the HPV vaccination. They felt that, ultimately, parents could still control their teenagers’ right to self-consent by choosing not to send them to school on the day of the HPV vaccination.

*‘…part of the school’s remit is to work with parents. I think it is to build up close relationships with guardians and parents. And to do something counter to that ethos might be very damaging.’ (Teacher 5)*

*‘I have no problem with children self-consenting*… *personally I ask the boys to talk to their parents*…*you are legally entitled to sign for yourself*, *but*…*your mum then will feel upset because you bypassed her and didn’t trust her enough to talk to her about it*. *So it is important to have that conversation*, *if possible*, *with parents or guardians*.*’ (Nurse 1)*

### 3. Design of the HPV education

This theme is subdivided into four further sub-themes: 1) Mandating HPV education through a spiral curriculum; 2) Content of HPV educational intervention; 3) Format of HPV educational intervention; 4) Receipt of HPV vaccine

#### 3.1 Mandating HPV education through a spiral curriculum

All participants spoke about various aspects of the design of HPV education. Almost all participants commented that they would like to see robust and consistent education in all post-primary schools related to all aspects of sexuality education including HPV and other STIs. They indicated that this education should include emotional, spiritual and health aspects related to sexual relationships. They agreed that education regarding HPV and other STIs should be a mandated component of the professional development curriculum supported by strong, clear teaching plans, taking responsibility away from individual teachers/nurses.

*‘It really has to be [mandated]. Because you can’t have people within the school opting out. You can’t disadvantage a group of children…I think it has to be that you are saying the same things to students, regardless of what their background is.’ (Nurse 3)*

They suggested a spiral curriculum where sexuality education is presented in each year of the curriculum with deepening levels of complexity.

#### 3.2 Content of HPV educational intervention

Participants suggested that introducing age appropriate information in year 10 (13–14 years old) would be a good option involving talking about general cancer acquirement rather than details of transmission routes. Conflicting opinions arose among teachers regarding the depth of detail that would be appropriate for year 11 (14–15 years old) students particularly around transmission routes associated with vaginal, oral and anal sex. Conversely, the majority of nurses felt particularly strong that this detail was essential in year 11 and may even be too late. Almost all the participants agreed that students in year 12 should be educated about all aspects of HPV including transmission routes associated with vaginal, oral and anal sex.

*‘Year twelve [is the right time]. I think at year eleven they are just… you can see them still babyish, nearly. I can’t think of another word to say it. Immature.’ (Teacher 4)*

*‘ I certainly believe that once you hit year twelve*, *you’ve missed the boat in so many*, *because they are sexually active by the time they hit year twelve*. *And they are not going in to buy condoms*. *They are not going anywhere to get free condoms*.*’ (Nurse 3)*

A few participants were unsure if talking about anal sex in an all-girls school would be acceptable to parents but agreed that if the context was discussing a homosexual relationship then they might consider that appropriate. Conversely, a number of participants highlighted the importance of discussing same-sex relationships with all students to ensure they are fully informed about transmission routes and appropriate protection.

*‘I’m just being honest…I do think there would be a certain amount of eyes raised if they found that you were talking about anal sex…which I don’t think they would do if you were in a mixed school, because a mixed school has men.’ (Teacher 4)*

*‘*…*we would have a lot of children with varied sexual orientation here in school*, *and I sometimes feel conscious that all I talk about is man/woman sex*. *We don’t really talk about female/female*, *male/male*. *And that needs to change*, *I suppose*, *among us all*, *too*.*’ (Teacher 9)*

They felt that HPV education should be taught alongside other STIs but also as a stand-alone subject to improve clarity as HPV has a preventative vaccination unlike other STIs.

#### 3.3 Format of HPV educational intervention

All participants agreed that face-to-face HPV education in school was the most apt approach and favoured a classroom setting with smaller group sizes. While participants indicated that a group of 6–15 would be ideal, they agreed that, due to resources, a class size of 20–30 students would be more practical.

Participants indicated that they felt that all students should be taught together (not separated by gender) as everyone can acquire HPV and develop illness and disease from HPV. They described the importance of mixing genders from an early age when educating students about sexual topics to normalise it and make it more comfortable–particularly for girls.

*‘We shouldn’t be hiding behind any sort of sexuality because it takes two people to have any sort of sexual relationship, be it same sex or opposite sex. So I think they should be educated together… with people that they are going to be sexually active with, because they do tend to be in those early years, within that grouping.’ (Nurse 3)*

However, three teachers (2 from single gender schools and 1 from mixed gender school) were unsure of mixing students of different genders as they felt that the students might be uncomfortable with that scenario. Several participants commented that it would be best if the students in the group were friends as they know and trust each other.

*‘I don’t know if you’ll get as many students being as comfortable in a mixed setting…they are definitely not going to talk about their own experiences in a big mixed setting. But again, because I teach in a single sex school, it’s more difficult for me to say that.’ (Teacher 2)*

All participants talked about the importance of not lecturing students but making the session short (30–120 minutes), factual and interactive to improve their attention and interest. Participants most commonly talked about using videos showing young females and males with real-life stories who have HPV. They indicated that young influencer/celebrity stories would be the most powerful, referencing the impact of Jade Goody’s story in the past [74].

*‘Jade Goody brought cervical cancer to the front, young girls all went to get their smears done, because a live person was there and said, look, I have had this and I am living proof.’ (Nurse 1)*

Other suggestions included the use of quizzes to help the students understand their gaps in knowledge to ‘shock’ them into that realisation. Facilitator/Student role playing or case studies were also mentioned by participants as effective teaching tools. The participants felt that adding practical elements into the lesson would enhance the learning including scientific experiments, smear model demonstration and condom demonstrations. They mentioned that, while it can be embarrassing at first for students, students value these types of practical exercises. Some commented that condom demonstrations could be controversial as a practical exercise in some schools due to parental attitudes.

*‘…it’s a wee bit embarrassing for them initially, but I think kids love those sorts of things because they go away thinking, oh but I could do that now if I had to. Especially the condom. They are mortified when it is put on the desk initially, but then actually you see them thinking, right, OK… yes, I could do that if I have to, or if I want to.’ (Teacher 9)*

Over half of the participants talked about the importance of providing an opportunity for anonymous questions during or after the education. Physical drop boxes in the classroom were deemed an apt method to enable the avoidance of ‘facetious’ questions. A post-education social media platform was also mentioned though concerns were raised about confidentiality. Text-a-Nurse was also deemed an apt option in schools without a nurse.

Participants had contrasting opinions regarding language used during this HPV education. Teachers favoured adhering to ‘safe’ medical terminology to maintain a professional environment. However, nurses, while still using medical terminology when talking to students, also indicated that students may feel more comfortable using their own terminology to ask questions and were supportive of that approach.

*‘I think medical terms are probably safer… it’s scary how many kids have access to things like porn hub these days… .and the sexualised language we all hear in class from boys that probably don’t understand really what it is… if you had a medical professional in, you maybe don’t want them going down this dark hole of all these kids talking in these horrible pornographic terms.’ (Teacher 1)*

*‘I think it would be good for me to always use the medical terms*, *but I think sometimes*…*for the pupils…to say penis or vagina is more difficult than to say*, *what if your balls hurt*, *rather than testicles*. *So I think*, *as long as it’s not offensive*. *They should know the correct terms but I think if they want to talk about it and it’s more comfortable to use the slang*, *then I don’t have an issue with that*.*’ (Nurse 5)*

Several participants indicated that supplementing this face-to-face HPV education with social media platforms would be useful as students are so attuned to these platforms.

*‘These kids are so TikTok plugged in… I even use TikTok sometimes in my class…I’ve even had a couple of girls at GCSE saying, TikTok is just so powerful for getting you to think. So I think…if there was a platform specifically designed and targeting young people, even with young people on it, would be really good.’ (Teacher 1)*

#### 3.4 Receipt of HPV vaccine

Almost all participants talked about the importance of providing students with the opportunity to receive the HPV vaccine after the education, indicating that most students would not approach healthcare providers outside of the school to receive the vaccine. Barriers identified by these professionals included lack of initiative, inability to drive to their GP, lack of time and reluctance to explain to their parents and/or GP why they want the HPV vaccine. Two nurses indicated that some students may go to a Family Planning clinic rather than their GP as this is a service that they, as nurses, often sign-post students to for sexual health matters.

*‘I think it would be great if you can teach them all about HPV and then also offer them a vaccine at the end of it. I think you would see a huge uptake from that. But if they had to go and hunt it out for themselves, I don’t think you would get too many.’ (Teacher 1)*

### 4. Delivery of the HPV education

Three subthemes arose within this theme; 1) Characteristics of an ideal facilitator of HPV education; 2) Teachers as facilitators of HPV educational intervention; 3) Nurses as facilitators of HPV educational intervention

#### 4.1 Characteristics of an ideal facilitator of HPV education

An overwhelming majority of participants indicated that external facilitators would be most suitable to teach HPV education. Delivery of this information by someone with a healthcare background was deemed important, with many participants, especially nurses, indicating that this is essential.

*‘Probably somebody with a nursing or medical background…Sometimes you feel like you are masquerading a little bit whenever you are just a teacher and you haven’t got real world experience…the realities of people who then have suffered from these different types of infections.’ (Teacher 11)*

*‘It has to be somebody who has the education behind them to take the questions that will be broader than the actual subject that you’re going to talk about*. *Because once you open that Pandora’s box into any sort of sexually transmitted disease*, *it just goes wider*.*’ (Nurse 3)*

However, all participants agreed that the personality of the facilitator was very important too. Participants talked about the importance of the facilitator being fun, open, informal, clear, non-judgemental and comfortable talking about the topic, and thought that a young person in their mid-twenties to mid-thirties, would be an ideal facilitator. The gender of the facilitators was not deemed to be of high importance and many rated co-facilitation with both genders together.

*‘The most important thing is that they feel you are going to talk to them in a relevant way. You’re not going to be judgemental with them. There has to be that trust…’ (Nurse 3)*

*‘I really*, *genuinely don’t think gender is an issue anymore*. *I think it’s the level of comfort from the young person really*, *I think is probably the most important*. *And I think for them*, *everything is so gender fluid now*.*’ (Teacher 7)*

Participants suggested that immunisation nurses, school-based nurses, healthcare students or ‘Love for Life’ teams would be ideal to deliver this teaching.

*‘…these guys rock in from Love for Life, they are really cool, they are age appropriate…they are in trendy clothes…and the kids relate to them.’ (Teacher 7)*

However, the majority of nurses indicated that HPV and other sexual health information should be delivered by, at minimum a band 5 nurse, in a specialised role.

#### 4.2 Teachers as facilitators of HPV educational intervention

Most teachers indicated that teachers would not be the most suitable facilitators of HPV education with the most cited reason being that teachers would be embarrassed and uncomfortable teaching this topic to their students. In addition, they felt that the students might not open up and ask questions, if they knew the teacher.

*‘…while they are difficult, awkward conversations, I think they are only difficult and awkward conversations for us. I don’t think for them they are…we are the ones who are carrying the baggage and the awkwardness and the… oh, I don’t want to talk about this!’ (Teacher 7)*

They felt that teachers did not have the requisite training and most ‘did not have a clue’ about HPV. Some teachers expressed a fear that students might use their phones to record them teaching this topic. Others indicated that the subject matter may not align with teachers’ beliefs.

*‘I do look at other teachers here in school who are maybe from a very religious background and I wonder if they would feel comfortable with actually discussing the fact that people have sex outside of wedlock! Honestly, we are still at that, like! Oh, it frightens the life out of me.’ (Teacher 9)*

*‘In our school you’d probably have a few teachers passing out*, *if you were mentioning words like that [oral sex]*! *(Teacher 10)*

They expressed concern about parental backlash from ‘saying the wrong thing’ when clear consistent guidelines were not provided. Subsequently, they felt that an external facilitator would be more suitable to deliver a mandated and standardised program in a manner that would be better for students.

*‘I think in many cases it comes down to whether the school is going to get a lot of backlash from parents… if it’s a situation where this is a decision that’s been taken out of the schools hands, this is mandated therefore we are just delivering what we have to deliver, that makes it a lot easier… .’ (Teacher 11)*

*‘*…*for me as a teacher*, *I would fear*, *if I’m talking about anal sex or oral sex*, *that for some reason that would provoke parents more than…that makes me a wee bit anxious*.*’ (Teacher 9)*

#### 4.3 Nurses as facilitators of HPV educational intervention

Nurses expressed their passion and love for their current role and for teaching young people about sexual health but indicated that adding this education to their current school role would not be manageable, as they would need to tend to illness/injury on demand which would make their teaching unreliable.

*‘I think the school based nurse would be good, because our relationship with the children is different…they expect us to be able to chat to them. Most of us wear a uniform, so it gives us that wee bit more health related stuff…we have good relationships with the majority of the children…it might be a bit freer to come and chat to us. So I think in an ideal world, it would be something that would be our role.’ (Nurse 5)*

Nurses demonstrated high levels of knowledge, experience and training regarding HPV and other sexual health issues. These nurses described helping students in their schools with suspected STIs and providing them with condom education. Only one nurse expressed concern regarding the teaching process itself and presenting information in front of students. All of the other nurses appeared to be confident in talking to the students in an open format.

Teachers indicated that behavioural management (particularly with boys) is a consideration when external providers are teaching this topic and suggested that a teacher might need to stay in the room to manage behaviour. However, they acknowledged that having a teacher present could negatively impact student openness during the teaching. They also felt unsure about external providers being alone, unaccompanied by a teacher, and stressed the importance of a disclosure policy. While nurses commented on potential differences in boys and girls behaviours, they did not express any concerns regarding their ability to manage students’ behaviour when facilitating sexual health education.

*‘If they think you are respecting them as young adults, they will behave like young adults.’ (Nurse 4)*

*‘*…*certain teachers also have varying strong views on certain things and that’s often apparent to students and the students might*…*maybe clam up and not want to ask questions of the nurse because of the teacher’s presence*.*’ (Teacher 1)*

## Discussion

Teachers and school-based nurses agree that the current HPV education provision in post-primary schools in Northern Ireland is inadequate to enable adolescents to make informed sexual health choices. They placed high importance on this education to increase HPV vaccine uptake, HPV screening and awareness of safe sexual practices to reduce transmission of HPV. They perceived HPV knowledge among adolescents to be poor and cited the main barriers as being a lack of parental education and cultural factors largely specific to Northern Ireland.

Northern Ireland has a unique political history and remains a deeply religious society consisting of mainly Catholics and Protestants [75]. Both Catholic and Protestant churches agree on many aspects related to the morality of sex, stressing abstinence before marriage and instilling a sense of shame and denial, particularly in regard to same-sex relations [76, 77]. In this study, teachers and nurses indicated that many parents are in denial about their child’s sexual behaviours and discourage the school from providing HPV and other STI education to adolescents under 15–16 years old, despite evidence from teachers and nurses that some students are sexually active younger than this age. Participants expressed fear of retaliation from parents when discussing sexual health issues with students and modified their content to what they perceived as ‘safe’. Previous studies which explored the impact of religious background on HPV vaccine acceptance in a variety of countries, reported inconsistent results [78]. However, parents who attend religious services frequently have been found to be more likely to not support HPV vaccination [78]. This highlights the care which must be taken by national health and education authorities to ensure that societal stigmatisation does not become a culturally acceptable form of regulation and control [79] in the delivery of post-primary school HPV education, but be approached rather as an important evidence-based health initiative.

Several teachers and nurses stressed the importance of working closely with parents to educate them and help them to have open conversations with their adolescent children. While these can be uncomfortable conversations, students who have a positive communicative relationship with their parents have been found to be less likely to initiate sex early and more likely to protect themselves during sex [80, 81]. Despite being a deeply religious society, there is a growing trend in Northern Ireland towards acceptance of sex before marriage [82], which participants also alluded to in this study. While pre-marital sex is growing even in traditional societies around the world, many countries including India, Indonesia and Iran deem pre-martial sex to be morally unacceptable [83], increasing barriers for open communication between adolescents and their parents.

In this study, participants perceived that anti-vaccination movements on social media platforms had contributed to the reduction in HPV vaccination uptake rates since the COVID-19 pandemic, consistent with other studies [84–86]. One study by Betsch et al. found that as little as 5 to 10 min of access to vaccine-critical websites influenced participants’ risk perception and vaccine intentions [84]. This strengthens the need for additional HPV vaccine education to help to dispel misinformation gained through social media platforms.

Participants agreed that HPV education should be embedded as part of a mandated consistent spiral sexual health curriculum with increasing details added to the education from year 10 through to year 12, though taught as a stand-alone topic. Conflicting views arose with regard to the timing of when to address transmission routes like vaginal, oral and anal sex with participants largely split between year 11 and year 12 being the right time. Some participants were strongly influenced by concern over parental response regarding talking about anal sex. Indeed, young people in Pound et al.’s review noted that sexual education in school is largely defined narrowly as heterosexual intercourse failing to acknowledge the full range of sexual activities that they engage in and want to discuss [82]. Globally, consistent with the findings of this study, sex education has historically focused on heterosexual intercourse with little to no sex education that reflects the experiences of gay, lesbian, bisexual and transgender adolescences [87, 88]. Subsequently, HPV education needs to be developed to ensure diversity, equity and inclusion to support all students’ sexual development.

One model of sexuality education, which has embedded these principals, is Comprehensive sexuality education (CSE) [89]. CSE has recently gained momentum throughout Europe and is increasingly being accepted as a model that enhances adolescents’ ‘capacity for informed, satisfactory, healthy, and respectful choices with regard to sexuality’ (p2) [89]. CSE promotes age and developmentally appropriate sexuality education in an incremental process that builds upon previous learning in a spiral-curriculum approach [89]. A core aim of this model is to promote positive student attitudes, reduce shame, anxiety and misinformation and break down gender assumptions. CSE builds on other sex-positive education models like that in Finland and the Netherlands, where this type of sex-positive education has contributed to lower levels of STIs, teenage pregnancy and abortion rates [89]. However, challenges exist in the delivery and assessment of CSE, particularly in developing countries [90]. These include consistency in delivery of the education due to lack of resources and/or time and teachers not adhering to recommended teaching practices [90]. Teachers’ motivation, attitudes and skills have also been found to be strongly linked to CSE success [90].

Participants highlighted the disparity in sexual activity among students in schools of different religious, socioeconomic backgrounds and genders, consistent with previous studies [91]. Participants in this study who advocated for early education regarding HPV transmission routes were concerned that vulnerable adolescents are receiving this information too late. Based on recent UK figures, approximately 3% of 14-year-old students and over 20% of 15-year-olds students engage in oral or penetrative sex [92–94], with those students who initiate sexual activity earlier being less likely to use contraception and more likely to contract STIs, like HPV [91]. To protect this group of young adolescents, many participants, particularly nurses, deemed detailed HPV and other STI education in year 11, when students are 14–15 years old, as essential. As the average age of sexual initiation differs considerably among counties [43], individual countries should tailor their education based on the average age of sexual initiation in their country.

Participants discussed the importance of providing another opportunity to receive HPV vaccination after the delivery of HPV education in year 12 and potentially year 11. Self-consent for the HPV vaccine was supported by the majority of participants for adolescents 15 and 16 years old. The majority of participants did not think that 14-year-old students would have the maturity to self-consent. As previously established, Gillick consent is a legal form of giving consent in the UK and a number of other developed countries, when adolescents are under the age of 16 years old [95–97] whereby a health professional assesses the adolescent’s understanding of the vaccination’s potential benefits and harms and they are deemed competent to understand. In this study, teachers were largely unaware of the concept of Gillick consent and some nurses were also unsure of the legal rights of students. This is consistent with previous studies, which highlighted confusion regarding Gillick consent among nurses and a reluctance to vaccinate adolescents under sixteen years old without parental permission [50, 66, 98, 99], Participants in Flood et al.’s [66] UK study also highlighted the importance of the child-parent relationship and reported that nurses were very reluctant to contravene parental wishes; similar to Gotvall et al.’s [99] Swedish study findings. The majority of participants were aware that 16 year olds can provide self-consent for vaccinations in accordance with UK law [96].

There was consistency in perceptions of the ideal format of the HPV education with participants agreeing that an interactive short class with small group sizes delivering key facts would be most effective. Embedded real-life experiences were cited most frequently as being impactful alongside practical demonstrations and opportunity to ask questions both openly and anonymously. This aligns with Pound et al.’s systematic review [87], which concluded that students enjoy smaller classes and group discussions where they can participate without being singled out. Active participation is key to empowering adolescents to gain capability of representing themselves and making their own decisions [89]. They also appreciated diverse activities including practical demonstrations [87].

The majority of participants favoured mixed gender HPV education though highlighted the importance of maintaining a respectful environment. Some teachers and nurses indicated that adolescent boys tend to be disruptive in sexual health discussions. A number of other studies also reported that adolescent boys were disruptive in sex education classes with some explaining their behaviour as a way of masking their anxiety [87]. Male adolescents communicate less with anyone, including friends and parents, about sex, relationships and condoms [80]. However, this negative behaviour has resulted in female adolescent students often preferring single sex education when the environment was not adequately controlled. This highlights the importance of ensuring that the right person is delivering this education and is confident controlling the dynamics of the classroom to improve the quality and benefits of this teaching.

Teachers and nurses indicated that young health professionals, particularly nurses, with open fun non-judgmental personalities, would make the best facilitators for this HPV education. Teachers were not deemed suitable mainly due to embarrassment teaching this topic and lack of training. This is consistent with previous global studies [87, 100] where students also agreed, regarding teachers as unsuitable for teaching sexuality education due to lack of training and embarrassment, which they perceived to affect the quality of the teaching [87, 100]. Teachers themselves commonly reported being embarrassed and awkward delivering this education [87] consistent with the findings of this study. Students in several studies [87, 100] reported that teachers seemed unable to discuss sex frankly. This is also apparent in this study as nurses spoke much more openly about HPV transmission routes and were experienced with talking to students about sexual health issues. Pound et al.’s review also reported that students perceived health professionals to be less judgemental and more informed than teachers [87].

Participants felt that teachers do not receive adequate training to teach HPV education to a high standard. Many teachers indicated that their HPV knowledge was poor with many being unaware of the link between HPV and male cancers. This knowledge gap is not uncommon among non-healthcare professionals [101, 102] and has been found to be particularly low in male teachers [102]. This reiterates the suitability of a health professional rather than a teacher for teaching HPV and other STI education. Most nurses expressed a strong desire to be involved with the design and delivery of HPV education.

## Implications

Health and education authorities should work collaboratively to mandate CSE which would include HPV education in post-primary schools, providing age appropriate information at all stages of this education; in Northern Ireland, these authorities are the Public Health Agency (PHA) and Education Authority (EA). Health professionals with expertise in this field should be recruited to design STI education including HPV education. This multilevel educational intervention should be grounded in behavioural theory [27] combining social media platform resources, interactional videos and other resources.

Evidence from this study suggests that motivated enthusiastic health professionals, possibly nurses, should be employed to provide this STI education within the CSE. These health professionals would ideally have a post-graduate qualification in public health or a similar relevant qualification. Consistent training would need to be provided which would include core teacher training, legislation guidance regarding adolescent consent and specific content delivery. Vaccination opportunity would need to be provided alongside any HPV education offered to students. Legislation should assure confidentiality for adolescents and provide legal protection for health care providers who administer vaccinations with informed consent.

Part of the education strategy should focus on educating parents, schools and religious institutions regarding the importance of HPV education and vaccination. Long-term goals for health authorities should include dispelling misinformation about HPV though social media by developing public health campaigns which promote vaccination safety and highlight the importance of HPV vaccination for both females and males.

## Study strengths and limitations

A strength of this study was that participants were recruited from a variety of types of schools throughout Northern Ireland. Given that the interviews were geographically limited to Northern Ireland, results from this study may not be generalizable to the whole of the UK. No inferences can be drawn about the prevalence of phenomena observed beyond the sample. Additionally, there was limited diversity within the study population with none of the participants describing themselves as belonging to a race or ethnic group other than white. In public health interviews, it is possible that interviewer characteristics can result in some bias in the data collected [103].

Ideally, piloting of the study would be conducted to address instrumentation and bias issues [64]. However, this was not practical due to the limited number of research participants available and the importance of including all of their valuable insights.

## Conclusion

While HPV vaccination in the UK is high, fluctuations in uptake are increasingly common. Additional HPV education may be beneficial to adolescents at a time when they are becoming increasingly sexually active. Teachers and school-based nurses place importance on mandated consistent HPV education at this time, delivered by a health professional who is open, fun and non-judgmental. HPV education should be short, interactive and address emotional, spiritual and health aspects in a safe comfortable environment. This additional HPV education has the potential to improve HPV uptake, HPV screening and sexual behaviours to reduce associated illnesses like cancer. The DoH and EA need to work collaboratively to provide guidance for schools to provide STI education through a mandated spiral curriculum as part of a larger sexual health initiative.

## Supporting information

S1 File Interview guide. (DOCX)
