# Supplementary material for: An exploration of teacher and school-based nurse perceptions of current HPV education offered to students 15–16 years old in post-primary schools in Northern Ireland, UK
Source: PLoS One. 2024 Oct 7;19(10):e0311651. doi: 10.1371/journal.pone.0311651 (PMC11458032; doi:10.1371/journal.pone.0311651)
Supplement: S1 File — (DOCX) [file pone.0311651.s001.docx]

**Semi-structured Interview guide for Teachers/Nurses**

**Key topic: Education**

The aim of this project is to find out whether you feel that any further education is needed regarding HPV vaccination, HPV transmission and risk for students who are 15-17 years old.

Q1. How do you feel about the current education provided in secondary schools for 15-17 students regarding HPV transmission, HPV vaccinations and HPV-associated cancers?

- Amount of information
- Appropriateness of information in year 12
- Timing of information – year 12
- Opportunity
- Perceptions of family and friends

**Key topic: Delivery**

Q2. In an ideal situation, if additional education were to be provided in the school setting, who should deliver information to year 12 students regarding HPV?

- Setting for information - Within/outside school (inconsistent in schools)
- People involved (internal/external to school; expertise)
- Psychological and physical capability
- Psychological motivation
- Format of delivery ; retention of information
- Barriers and facilitators to HPV education programme and vaccination
- Overcoming barriers
- Part of broader sexual health programme
- Separate groups of genders or together

**Key topic: Content**

We would like to know what kind of information you think should be provided to these students to help them to make informed decisions about HPV vaccination and sexual health related to HPV.

Q3. What content do you think should be included in a HPV education programme in year 12?

- Risks and transmission routes
- HPV vaccination protection
- Opportunity to get HPV vaccination
- Barriers and facilitators to receiving vaccine
- Consent
- HPV Screening – would they attend based on their current knowledge?

**Key topic: Implications**

Q4. What impact do you think this HPV education might have on year 12 students and their future health?

- Not providing education
- Providing education
- Goals of intervention
- Monitoring HPV uptake
- Benefits of getting additional HPV information
- Ability to impact sexual health behaviour
- Societal impact
